# Supplementary figures and images for: CLARITY increases sensitivity and specificity of fluorescence immunostaining in long-term archived human brain tissue
Source: BMC Biol. 2023 May 24;21:113. doi: 10.1186/s12915-023-01582-6 (PMC10207789; doi:10.1186/s12915-023-01582-6)

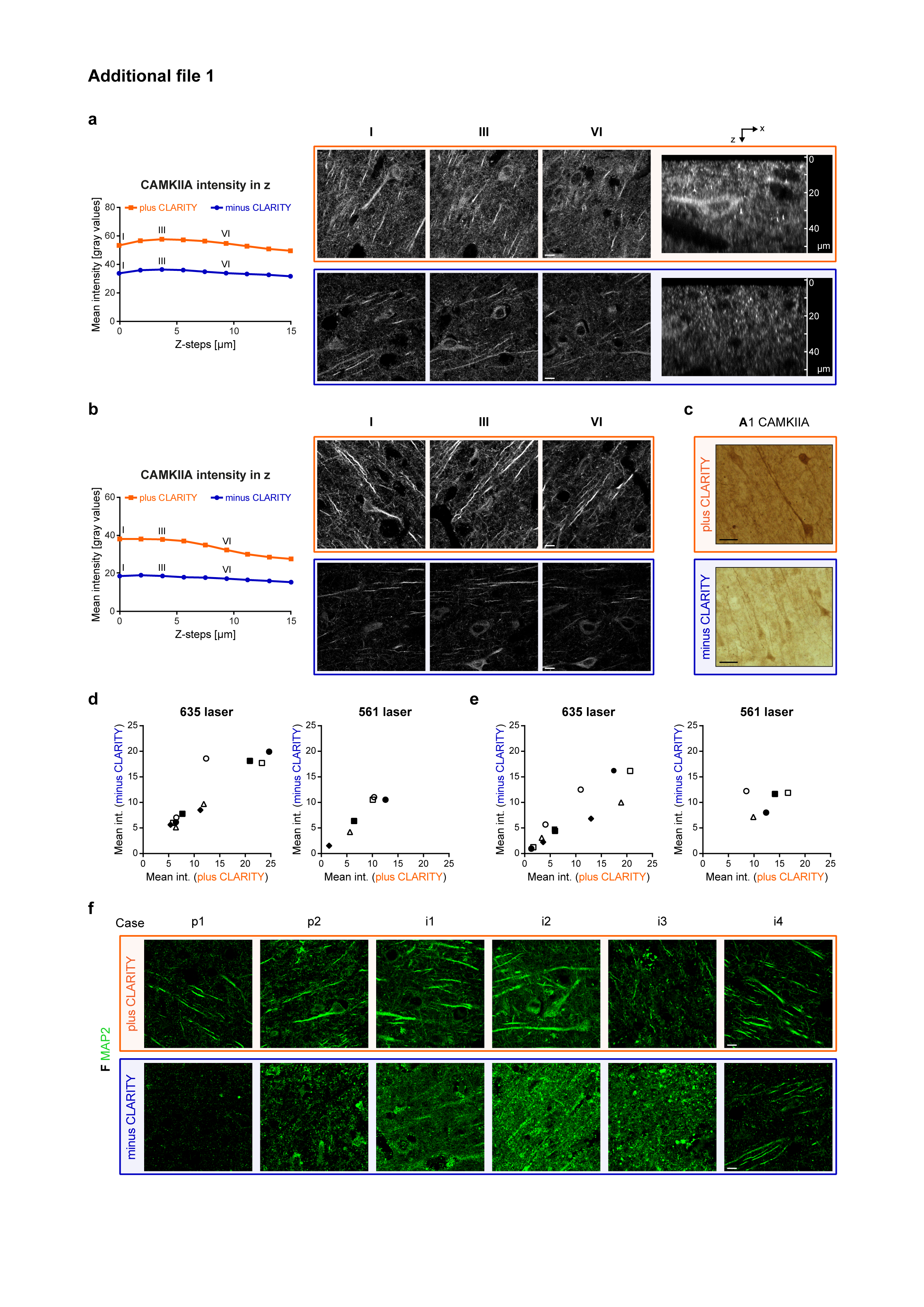

Supplement: Supplementary file 1 — Additional file 1. Improved antibody labeling and better comparability between different cases with CLARITY. (a) Cleared (orange) and non-cleared (blue) frontal cortex sections (superior frontal gyrus) from case i4 (total fixation time 18 years) were stained for CAMKIIA and imaged 15 µm deep in 1.8 µm steps using a confocal microscope. Mean intensities of each z-step were exported with LAS-X software from two stacks (cortex layer V) and the mean was plotted in dependency of the z-level. Z-level I (z = 0 μm), III (z = 3.6 μm), and VI (z = 9 μm) are provided as single images. A side view (xz scan) is shown on the far right. Scale bars, 10 µm. (b) Mean intensity of CAMKIIA staining on case i1 (total fixation time 5 years) was plotted in dependency of the z-level. Acquisition strategy is the same as outlined in panel (a). Scale bars, 10 µm. (c) CAMKIIA staining with DAB on cleared (orange) and non-cleared (blue) sections (case p2). Images represent full focus projections of z-stacks with identical brightness/contrast adjustments. Scale bars, 20 µm. (d) Background levels in secondary antibody only controls do not differ with CLARITY. Graphs show the mean intensities (int.) of the negative controls (secondary antibody only) of minus and plus CLARITY tissue sections; each case is represented by a different symbol (● i1, □ i2, ○ i3, ■ i4, ♦ p1, and Δ p2). The mean intensity of the entire image was determined in ImageJ. Negative control data belonging to CAMKIIA-rb, GLUA2-ms (both imaged using the 635 laser), and MAP2-ms staining (561 laser) are shown. Negative controls were always acquired with identical settings for both conditions (plus/minus CLARITY), mostly corresponding to the staining (in few cases higher laser power), which included the primary antibodies (see Figs. 2 and 3). (e) Second technical replicate. 561 laser: case p1 was excluded since negative controls acquired with identical settings were not available. (f) Comparison of MAP2 labeling on cleared (orange) a [file 12915_2023_1582_MOESM1_ESM.tif]

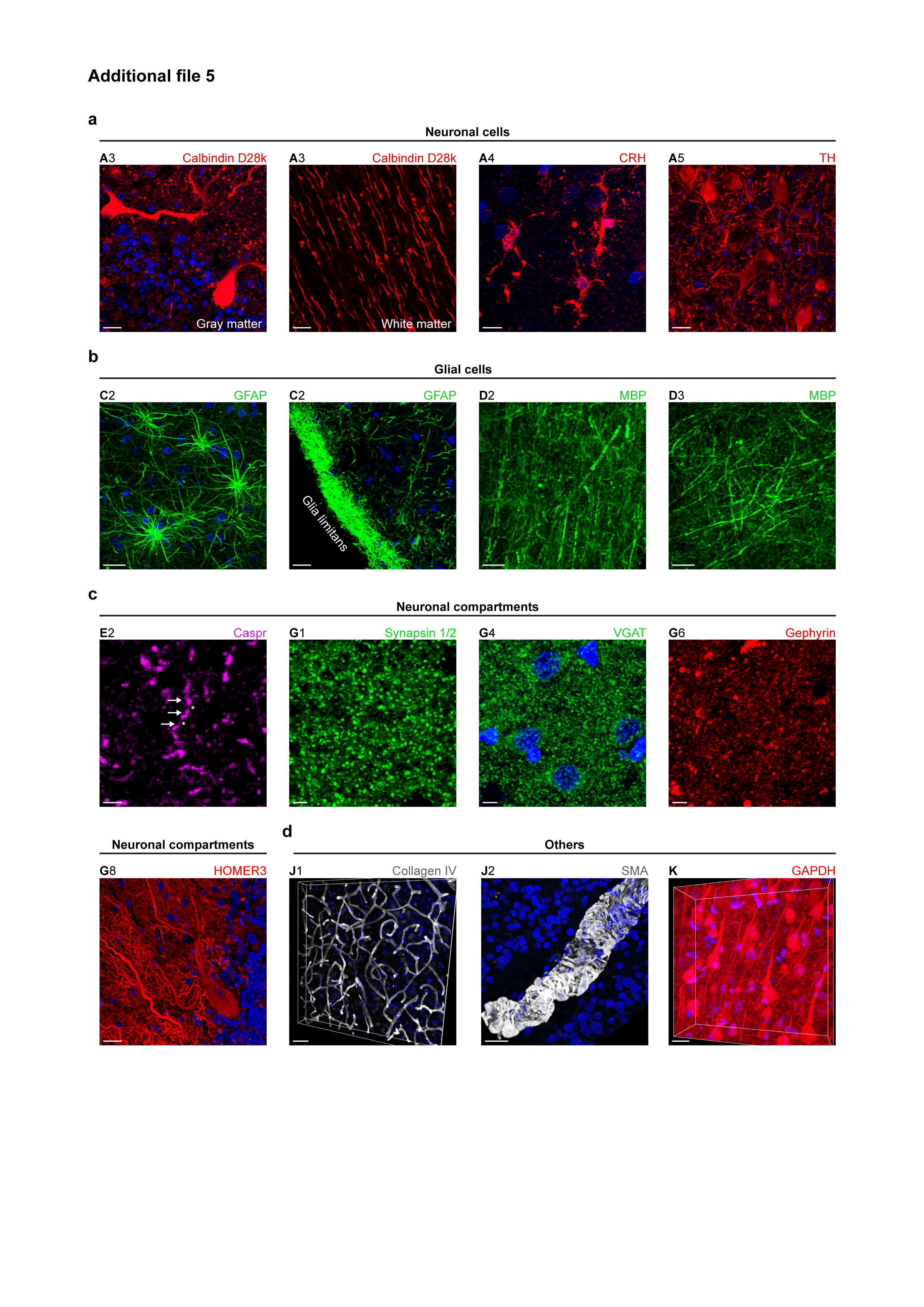

Supplement: Supplementary file 5 — Additional file 5. Additional CLARITY-compatible antibodies on human brain sections. (a) Neuronal cell markers used to detect specific neurons in cerebellum (A3), hippocampus (A4), and locus coeruleus (A5). Scale bars, 20 µm (A3), 10 µm (A4), 30 µm (A5). (b) Glial cell markers used on frontal cortex sections. Scale bars, 20 µm (C2), 30 µm (D2, D3). (c) Neuronal compartments, e.g., paranodes of axons (E2, exemplarily marked with arrows, asterisks mark nodes of Ranvier), presynapses (G1, G4), and postsynapses (G6, G8) are highlighted in frontal cortex sections (E2, G1), putamen (G4), pallidum (G6), and cerebellum (G8). Scale bars, 3 µm (E2), 5 µm (G1, G4, G6), 20 µm (G8). (d) Vessel-related markers (J1, J2) and GAPDH (K) in cortical sections. Scale bars, 50 µm (J1), 20 µm (J2, K). DAPI counterstaining is depicted in blue. All images except A3 (both) and C2 (right) represent MIPs of z-stacks, K is shown in perspective view. Staining is shown for perfusion-fixed cases (mostly p1 and p2, except A3, G4, and J2). The upper-case letter at the top of each image refers to the entities outlined in Fig. 5a and the combination of letter and number allows for identification of the applied antibody listed in Table 2. [file 12915_2023_1582_MOESM5_ESM.tif]

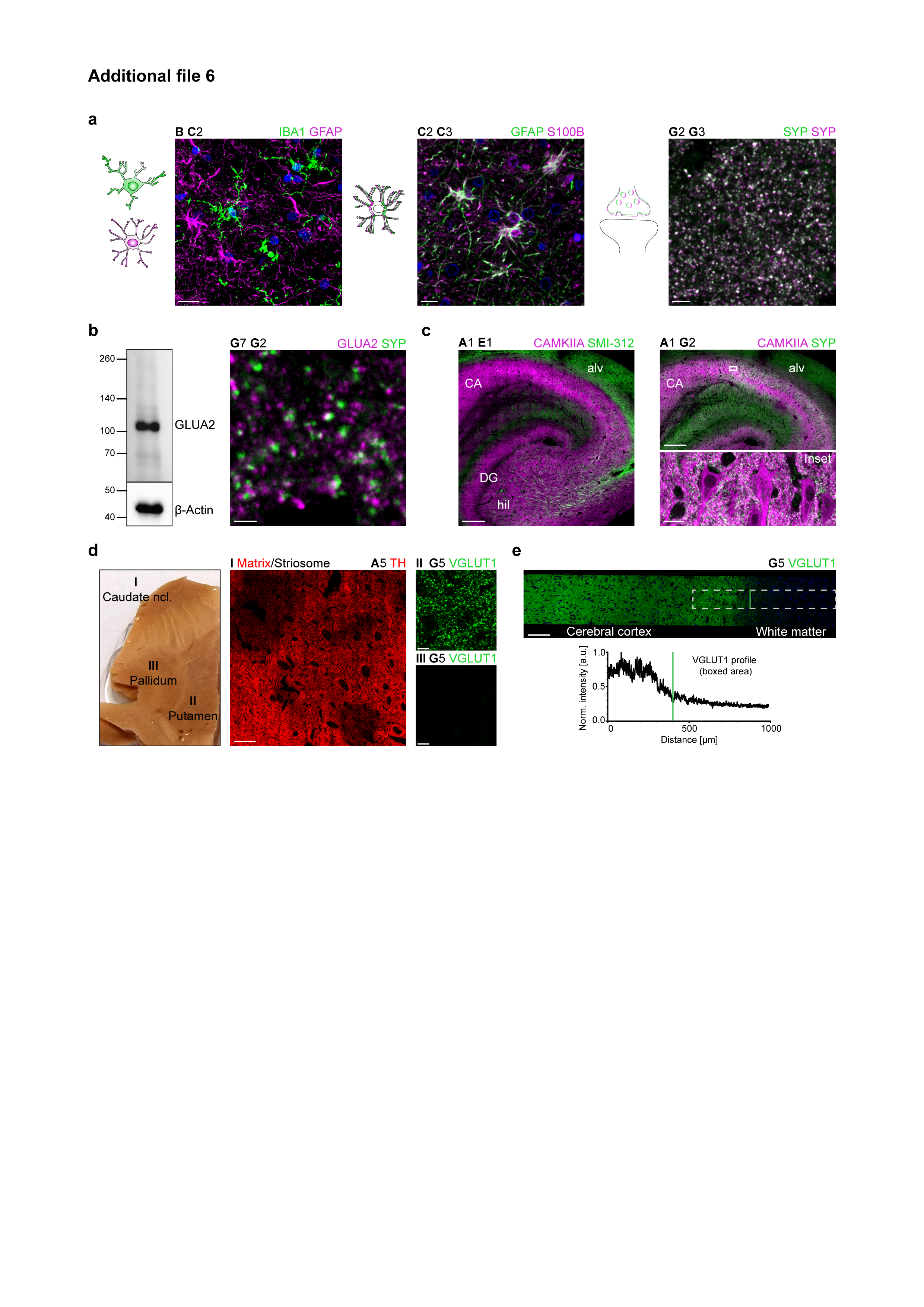

Supplement: Supplementary file 6 — Additional file 6. Cell, compartment, and region specificity of applied antibodies with CLARITY. (a) Co-staining with glial cell markers and two presynaptic markers as proof-of-specificity. DAPI counterstaining is shown in blue. Scale bars, 15 µm (left), 10 µm (middle), 5 µm (right). Images are mainly derived from frontal cortex sections of cases p1 and p2 (C2 C3: underlying white matter). Except C2 C3, images represent MIPs of z-stacks. White color indicates overlap of markers. (b) GLUA2 (G7) was used for western blots with human cerebral cortex lysate (Protein Medley, 50 μg), exposure time was 30 min for GLUA2 and 30 s for β-actin (original blot: Additional file 17). Unit of the ladder is kilodalton. The same antibody is shown in a synaptic staining on frontal cortex sections (case p2). Scale bar, 2 µm. (c) Triple-staining with neuronal, axonal, and synaptic markers in a hippocampal section (case p2). For better visualization CAMKIIA is shown twice, in combination with SMI-312 (left) and with SYP (right). For the latter, an inset of the CA is provided in higher magnification (scale bar, 20 μm). CA = cornu ammonis, DG = dentate gyrus, alv = alveus, hil = hilus. Scale bars, 500 µm. (d) Tissue block with striatum (I, II) and pallidum (III). Immunolabeled sections from this block show matrix (TH high) and striosome (TH low) compartments in the caudate nucleus (ncl.) (I). VGLUT1 expression is strong in putamen (II) and absent in pallidum (III). G5 images represent MIPs of z-stacks. Scale bars, 400 µm (TH), 10 µm (VGLUT1). (e) VGLUT1 expression in the superior frontal gyrus and white matter (top). Intensity profile of the dashed area (raw data), normalized (norm.) to the maximum intensity (bottom). The green line marks the gray/white matter border, which was approximated by the abrupt reduction of the VGLUT1 signal. a.u. = arbitrary unit. Scale bar, 150 µm. Sections in (d) and (e) were derived from cases p1 and p2. The upper-case letter at the top of each image refers t [file 12915_2023_1582_MOESM6_ESM.tif]

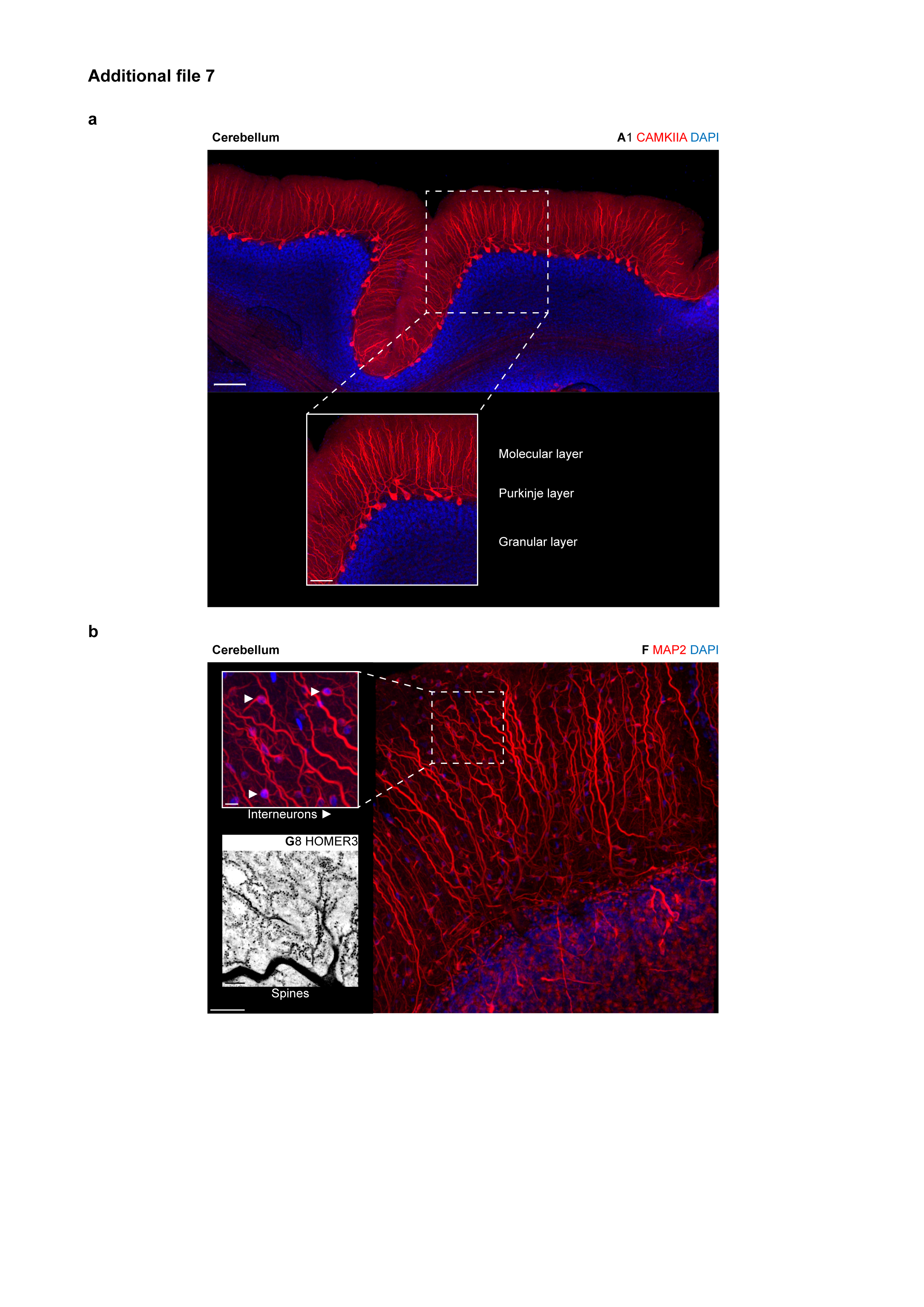

Supplement: Supplementary file 7 — Additional file 7. 3D tilescans of neurocellular and synaptic markers in the human cerebellum after CLARITY. (a) CAMKIIA in the cerebellum, MIP of stack with x ~3.2 mm, y ~1.5 mm, and z = 54 µm. Scale bars, tilescan 200 µm, inset 100 µm. Stainings were performed on case p1. (b) 3D tilescan showing MAP2 and HOMER3 (shown as inverted image to highlight the spines of Purkinje cells in the molecular layer) in the human cerebellum, MIP of stack with z = 63 µm (MAP2). Scale bars, tilescan 50 µm, insets 10 µm (top), 5 µm (bottom). [file 12915_2023_1582_MOESM7_ESM.tif]

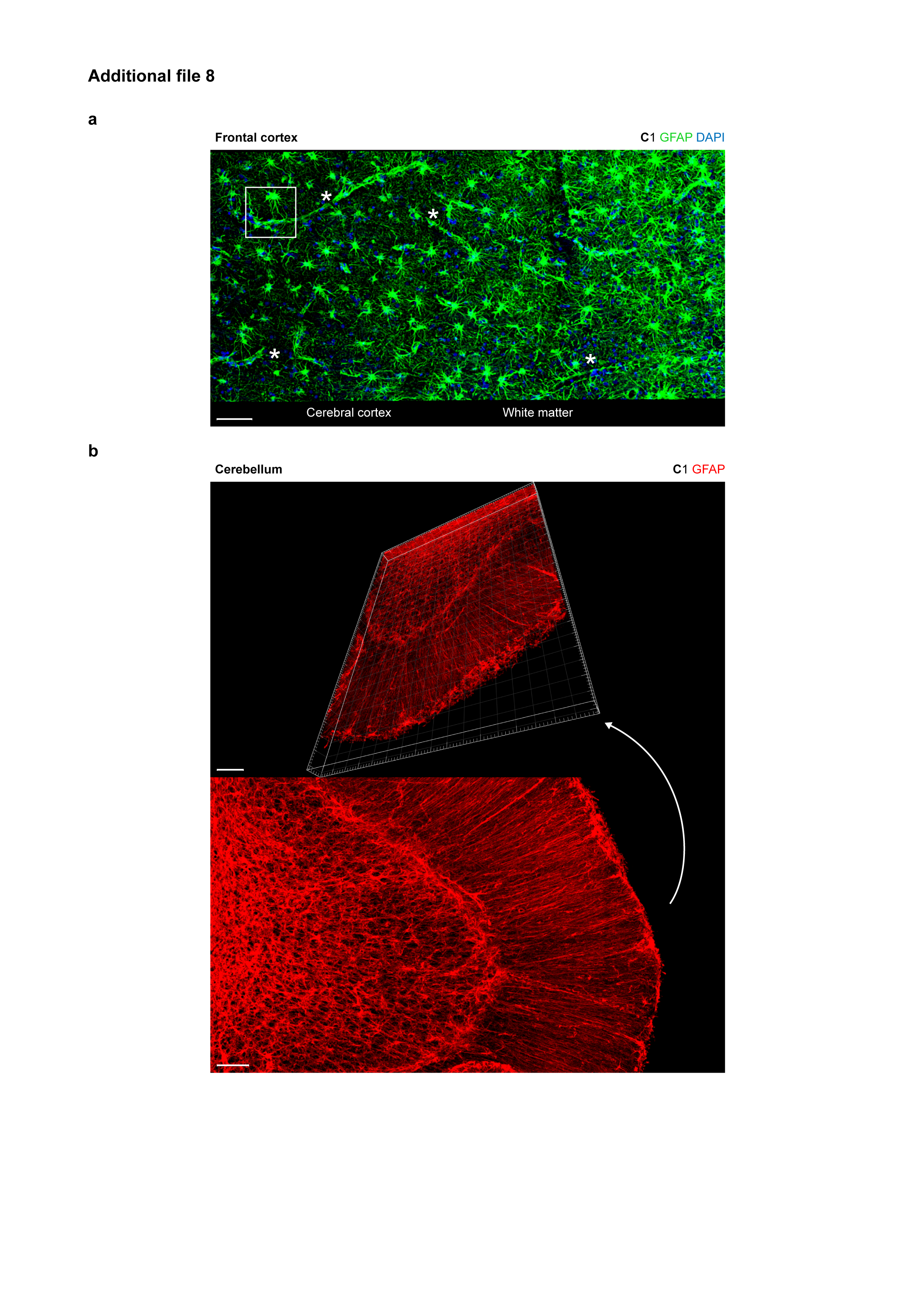

Supplement: Supplementary file 8 — Additional file 8. 3D tilescans unveiling the extensive meshwork formed by GFAP-immunoreactive fibers after CLARITY. (a) Cerebral cortex and adjoining white matter stained for GFAP, MIP of stack with z = 10 µm is shown. Asterisks mark several sites where the blood-brain barrier can be recognized by GFAP-positive extensions wrapping the voids where a vessel runs along. The boxed area is shown in higher magnification in Fig. 5b. Scale bar, 70 µm. (b) Cerebellar section stained with GFAP; dimensions in xyz, x = 848 µm, y = 1.1 mm, z = 56 µm. Scale bars, 100 µm (top), 70 µm (bottom). Sections were derived from perfusion-fixed cases (for panel (b) case p1). [file 12915_2023_1582_MOESM8_ESM.tif]

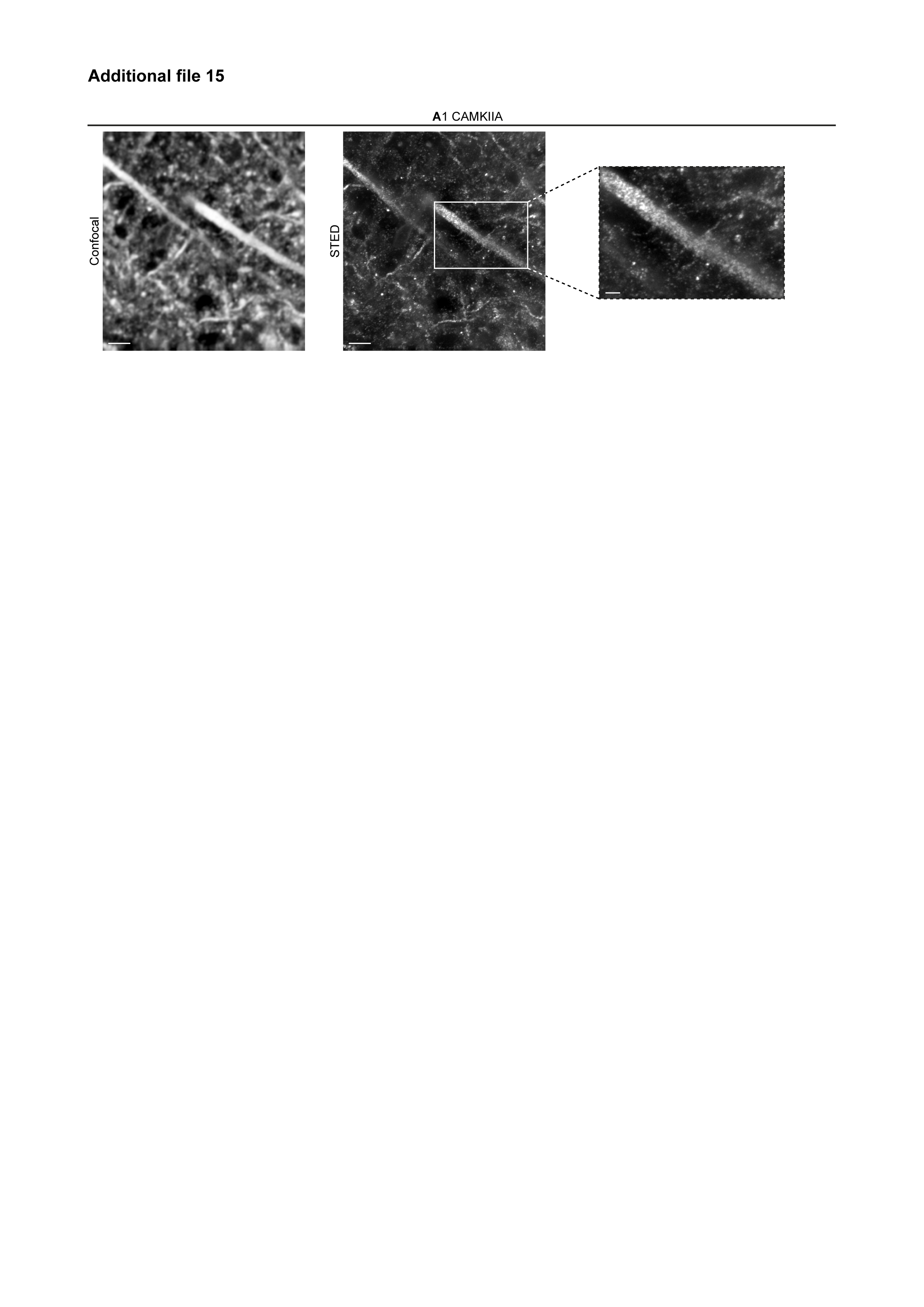

Supplement: Supplementary file 15 — Additional file 15. STED microscopy of CAMKIIA in human tissue after hCLARITY. CAMKIIA signal was acquired in confocal (left) and in STED mode (middle and right). Images are shown as MIPs of a stack with z = 3.6 µm. Z-drift was corrected with the Huygens Object Stabilizer (SVI). A higher magnification of a dendrite is shown on the right. Scale bars, 3 µm (left and middle), 1 µm (right). Pixel size for this confocal image is 100 nm, sections were incubated in hydrogel solution for seven days. [file 12915_2023_1582_MOESM15_ESM.tif]

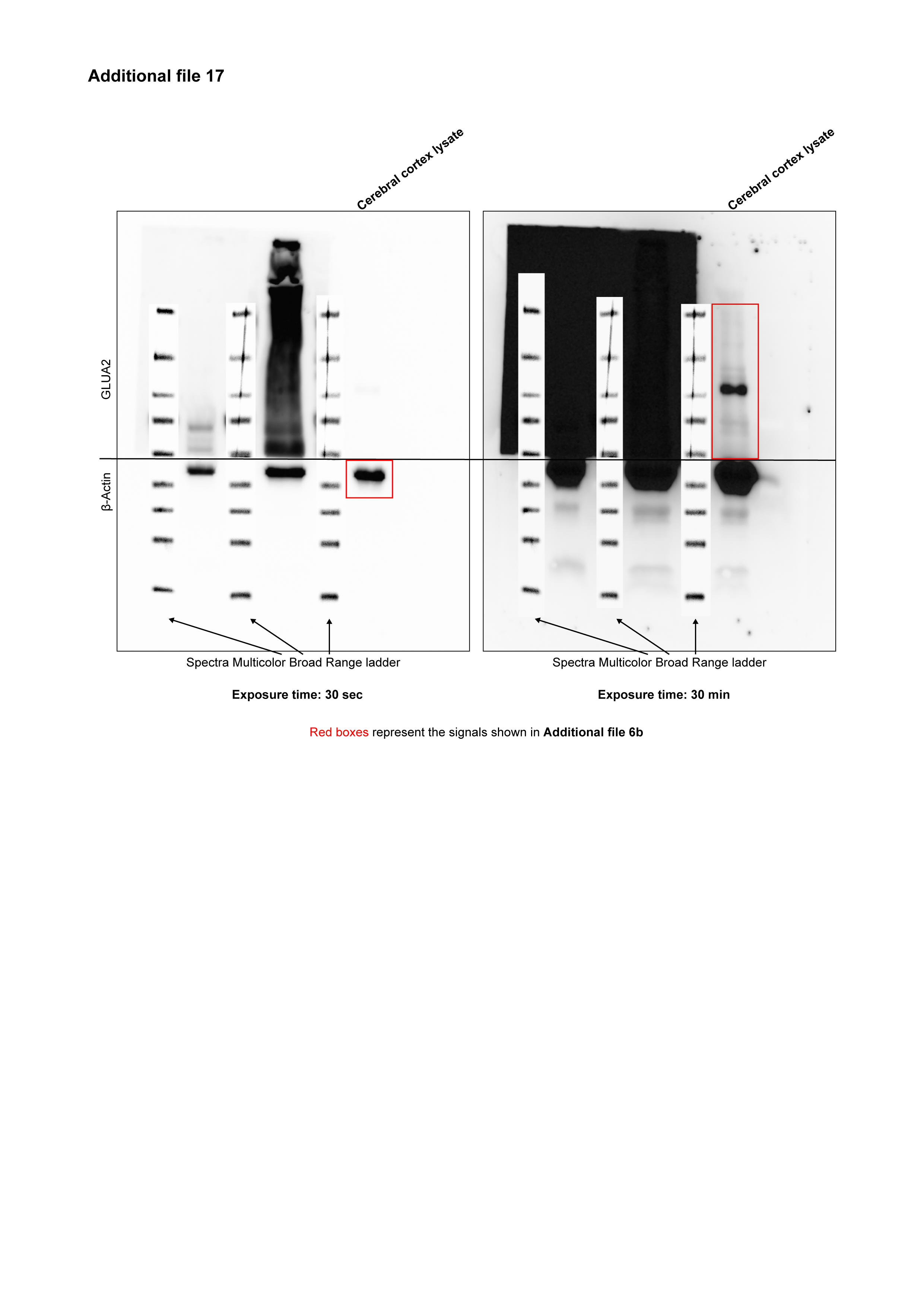

Supplement: Supplementary file 17 — Additional file 17. Original blot for Additional file 6b. [file 12915_2023_1582_MOESM17_ESM.tif]
